# Macro and microscopic comparison of the upper pole of the spleen with the lower pole in partial splenectomy^1^

**DOI:** 10.1590/s0102-865020200090000002

**Published:** 2020-10-16

**Authors:** Lucas Nagib Lemos Paulo, Daniel Robert Alexander, Elisa Ines Demuner Vallandro, Raquel de Azevedo Benevides, Luciene Lage da Motta, Marcela Souza Lima Paulo, Danilo Nagib Salomão Paulo

**Affiliations:** I Graduate student, School of Medicine, Escola Superior de Ciências da Santa Casa de Misericórdia de Vitória (EMESCAM), Vitoria-ES, Brazil. Scientific and intellectual content of the study, technical procedures, manuscript preparation and writing.; II Graduate student, School of Medicine, EMESCAM, Vitoria-ES, Brazil. Scientific and intellectual content of the study; technical procedures; acquisition, analysis and interpretation of data.; III Veterinarian, EMESCAM, Vitoria-ES, Brazil. Technical procedures.; IV Full Professor, Department of Pathology, School of Medicine, EMESCAM, Vitoria-ES, Brazil. Histopathological examinations.; V PhD, Associate Professor, School of Medicine, Animal Research Laboratory, EMESCAM, Vitoria-ES, Brazil. Critical revision, final approval.; VI PhD, Full Professor, Department of Surgery, Animal Research Laboratory, EMESCAM, Vitoria-ES, Brazil. Scientific, intellectual, conception and design of the study; statistics analysis; manuscript preparation and writing; critical revision; final approval.

**Keywords:** Spleen, Splenectomy, General Surgery, Rats

## Abstract

**Purpose:**

To evaluate the viability of the upper (UP) and lower pole (LP) of the spleen from a macro and microscopic point of view, after subtotal splenectomy with preservation (SSP) of the UP and the LP.

**Methods:**

Seventeen male Wistar rats, two months old, were submitted to SSPUP and SSPLP and 5 to simulated operation (SG). After 80 days, the rats were euthanized, and the remaining LP and UP and intact spleens were evaluated macroscopically and microscopically.

**Results:**

Two rats died during the operation. Macroscopic analysis showed that in 15 LP, one of them was not viable and in 15 UP and in 5 spleens in the SG, all were viable. In the statistical analysis, there was no difference in relation to viability. The LP and UP analyzed showed variation. As for the length, the UP increased significantly; however, in relation to the width, there was a significant increase in the LP in relation to the UP. In addition, the weight of the UP was significantly greater than that of the LP. Microscopic analysis attested viability of the splenic remnants.

**Conclusion:**

There was no significant difference regarding the viability of UP and LP, in macroscopy and microscopy.

## Introduction

Over the years, several studies have demonstrated the importance and usefulness of the spleen on the physiological functioning of the human organism. The two most important activities of the spleen in man, the immune and phagocytic functions, are determined by cellular composition, richness of irrigation and peculiar structure. Due to the recognition of its important immune function, surgical treatment of total splenectomy is increasingly avoided. For this reason, partial splenectomy has been performed for an increasing number of indications^1^. In addition to these primordial functions, it was demonstrated in an experimental study that the spleen has a role in the lipid metabolism of rats^2^. This result has not been demonstrated in mice^3^.

More recently, partial splenectomies have been performed safely and effectively by laparoscopy, by experienced surgeons and assisted robotics with good results and a rare conversion rate^4,5^.

It is known that the spleen is the solid organ most affected in closed abdominal trauma. In this situation, conservative treatment can be reserved for certain cases, guided by the complementary exam (tomography, doppler ultrasound)^6^.

In 1988, Petroianu^7^ published an article exposing the subtotal splenectomy technique preserving the upper pole, irrigated exclusively by splenogastric vessels, in the treatment of portal hypertension. In 1998, Resende and Petroianu^8^ published about the treatment of severe spleen injuries with partial splenectomy, reiterating the importance of this organ in preventing future infections and sepsis. More recently, this author published a study on the preservation of the upper pole in a patient with chronic lymphocytic leukemia^9^.

Paulo *et al*.^10^ published a study on subtotal splenectomy in dogs, with preservation of the lower pole supplied by vessels of the gastroesplenic ligament, evaluating the viability of the lower pole of the spleen of dogs, after ligation and section of splenic artery and vein. More recently, this technique has been the subject of a review study^11^. A more current study still deals with subtotal splenectomy with preservation of the lower pole in patients with Hodgkin’s lymphoma^12^. Other studies on subtotal splenectomy with preservation of the lower pole in last five years have been published^13-14^.

However, there are no studies in the literature comparing the upper pole of the spleen with the lower pole after subtotal splenectomy surgery. Based on this information, the present study aims to assess the upper and lower pole of the spleen from a macro and microscopic point of view, after splenectomy of the middle portion of the spleen.

## Methods

This project was approved by the Ethics Committee on the Use of Animals (CEUA-EMESCAM), under protocol number 003/2018. The handling and manipulation of experimental animals followed Law No. 11.794-2008, which regulates the use of animals in Brazil and the CONCEA Normative Resolutions regarding the use of animals in research activities.

Twenty-two male Wistar rats (Rattus norvegicus), two months old, were randomly distributed (sealed and closed envelopes) in two groups: 1 - simulation (n = 5); 2 - experiment (n = 17). The animals came from a *bioterium* accredited by the National Council for the Control of Animal Experimentation (CONCEA). They were allocated in collective cages, with up to three animals in each. The cages were kept in *bioterium*, inside cabinets, with temperature control, ventilation and light. They were fed with food for laboratory animals and filtered water in all phases of the experiment.

### Surgical procedure

**Figure 1:**
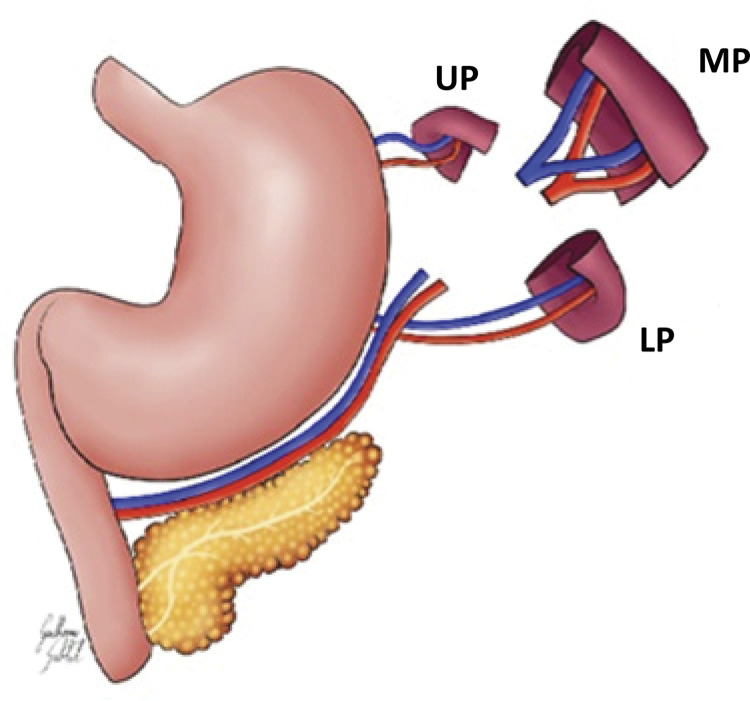
Schematic drawing of the surgery used in this study. UP: upper pole, LP: lower pole, MP: medium portion removed.

The animals were anesthetized with a mixture of ketamine hydrochloride (75 mg/kg of weight) and Xylasina hydrochloride (2.5 mg/kg of weight) injected intraperitoneally, after immobilization of the rat. Next, trichotomy and antisepsis of the abdominal wall were performed with a 2% iodized alcohol solution and placement of a fenestrated surgical field. In sequence, a median laparotomy, about 2.5 cm long, and examination of the abdominal cavity was performed. In the simulation group, only the spleen was manipulated. In the experiment group, the middle portion of the spleen with its supply vessels was removed, maintaining the upper pole and the lower pole of the organ in the same animal according to the techniques recommended by Petroianu^7^ and Paulo^10^, respectively (Fig. 1). Each pole was evaluated for length, width and thickness with a digital caliper.

The medium portion removed was placed in a bottle with 10% formaldehyde. Then, the abdominal wall was sutured in two suture planes: 1st plane - suture of the aponeurosis with the peritoneum, simple suture, mononylon 5.0; 2nd plane - skin suture, simple suture, mononylon 5.0. In the immediate postoperative period, the animals received dipyrone at a dose of 200mg/kg, orally, dissolved in drinking water, free diet and water at will. It was also used Nubain 0.1mg/kg of weight applied to the subcutaneous, 12/12 hours for 3 days. The animals that died were necropsied, and those that survived were killed on the 80th postoperative day, for macro and microscopic evaluation of the upper and lower poles of the spleen and measurement of their length, width and thickness.

### Euthanasia of animals

The animals were killed with an overdose of sodium pentobarbital injected into the abdominal cavity. After confirmation of respiratory arrest, dose-effect potassium chloride was administered intracardiac. They were weighed and submitted to an inverted “U” incision in the abdominal wall, for opening and examining the cavities.

### Macroscopic evaluation of the spleen and spleen poles

After looking at the abdominal cavity, the appearance, color, consistency, size and presence or absence of fibrosis and necrosis of the upper and lower pole of the spleen were verified, which were again evaluated for their length, width and thickness. Then, the respective spleens from the simulation group and the poles from the experimental group were removed. The removed organs were weighed on a precision scale, evaluating the macroscopic aspect.

### Microscopic evaluation of the spleen and its poles

Regarding microscopic evaluation, the simulation group and the middle portion of the spleen were placed in 10% formaldehyde solution, while the upper and lower poles, removed on the 80th PO day, were included in paraffin blocks and cut in a rotating microtome with three micrometers thick. Histological sections were placed on glass slides and in an oven at 58ºC for 24 hours. Adhered to the slides, these cuts were dewaxed in xylol and stained using Masson’s hematoxylin-eosin (H&E) and trichrome methods. Microscopy was performed by two pathologists using a binocular microscope. The cuts were analyzed for the following parameters: lymphatic follicles (germinal centers), number of lymphocytes, number of sinusoids, cell proliferation, number of vessels, fibrosis and necrosis.

### Statistical analysis

The following statistical methods were used:

- Descriptive statistics to calculate the arithmetic mean, standard deviation and median of the following variables: rat weight, spleen weight, upper and lower pole weight, middle portion weight, length, width and thickness of the upper and lower pole and the middle portion.
- Student’s t test or Wilcoxon’s non-parametric test for related samples, to compare the initial length with the final length, the initial width with the final width and the initial thickness with the final thickness, as well as the initial weight of the rats with the final weight.
- Independent student t test or Mann Whitney U test to compare the size difference between the two poles.
- The normality of the variables was analyzed using the Kolmogorov-Smirnov test.
- p ≤ 0.05 was considered statistically significant.
- Data analysis and statistics were performed using SPSS version 23.

## Results

In the course of the experiment, two animals died shortly after anesthetic induction.

The average weight of the rats increased from the beginning to the end of the experiment (80 days) from 326.13 ± 36.12 to 501.60 ± 37.52 (p <0.0001). At the end of the experiment, the weight of the upper pole was significantly greater than the weight of the lower pole (Table 1).

**Table 1:** Weight of splenic poles at the end of the experiment.

Upper pole			Lower pole			p

Minimum	Maximum	Median	Minimum	Maximum	Median	
159.70	392.60	250.00	0.00	400.30	161.50	0.0013

Mann Whitney U test. p≤0.05 significant

**Figure 2:**
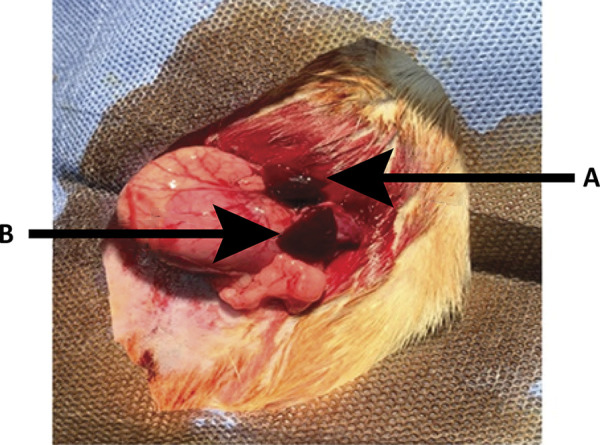
- Immediate result of removal of the middle portion of the spleen in rats, with preservation of the upper and lower poles. A. Upper pole; B. Lower pole.

**Figure 3:**
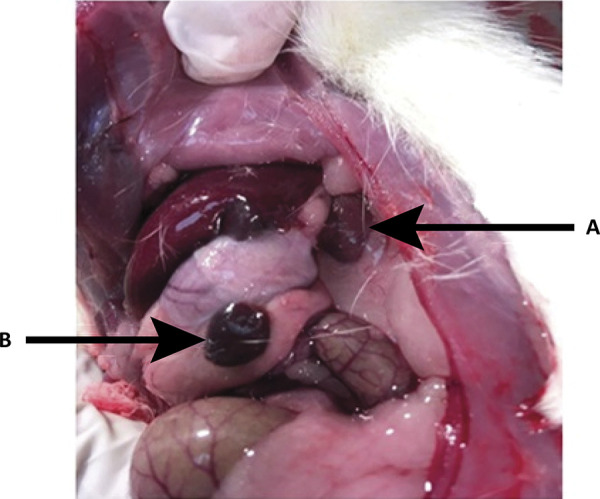
Late result, 80 days after removal of the middle portion of the spleen with preservation of the upper and lower poles. A. Upper pole; B. Lower pole.

The results of the measurements of the upper and lower pole before and at the end of the surgery are shown in Tables 2 and 3. Macroscopic aspects (viability) of the upper and lower pole of the spleen after subtotal splenectomy with removal of the middle portion are shown in Table 4, and in Figures 2 and 3.

**Table 2:** - Length, width and thickness of the splenic poles at the beginning and end of the experiment.

	Upper pole					Lower pole				
	
	Initial		Final		P	Initial		Final		P
		
	MA	DP	MA	DP		MA	DP	MA	DP	
L	7.46 ± 1.27		10.56 ± 1.56		<0.0001	8.02 ± 1.01		8.06 ± 2.65		0.83
W	7.13 ± 1.04		7.26 ± 1.17		0.70	8.30 ± 0.99		7.73 ± 2.51		0.30
T	3.90 ± 0.98		4.30 ± 0.75		0.21	3.70 ± 0.45		3.60 ± 1.33		0.76

L – Length, W - Width, T - Thickness. MA - Mean Arithmetic. SD - Standard Deviation. Student’s t-test for related samples. p <0.05 significant.

**Table 3:** - Difference in length, width and thickness of the upper and lower pole at the beginning and end of the experiment.

	Upper pole x Lower pole													
	
	Initial UP			Final LP			P							p
		
	Initial UP			Final LP				Initial UP			Final LP			
	Min	Max	Median	Min	Max	Median		Min	Max	Median	Min	Max	Median	
L	5.00	9.50	7.00	6.50	10.00	8.00	0.14	7.00	12.00	11.00	0	11.00	9.00	0.004
W	5.00	9.00	7.00	6.50	10.00	8.00	0.007	5.00	9.00	7.00	0	11.00	9.00	0.026
T	2.50	6.00	4.00	3.00	4.50	4.00	0.64	3.00	5.00	4.00	0	5.50	3.50	0.115

UP - Upper Pole, LP - Lower Pole. L – Length, W - Width, T - Thickness. Mann Whitney test. p≤ 0.05 – significant.

**Table 4:** Macroscopic aspects (viability) of the upper pole and the lower pole of the spleen after subtotal splenectomy with removal of the middle portion in 15 rats and with preservation of the entire spleen in 5 rats.

Group	N	Normal	Changed	p^1^	p^2^	p^3^	p^4^	p ^5^
UP	15	15	0 (100%)					
LP	15	14	1 (6.6%)	1.00				
MP	15	15	0 (100%)		1.00		1.00	
SG	5	5	0 (100%)			1.00		1.00

UP - Upper Pole, LP - Lower Pole, MP - Middle Portion, SG - Simulation Group. p1 - UPxLP Group; p2 - UPxMP Group; p3- Group UPxSG; p4 - Group LPxMP; p5 – LP xSG.

Note: The number of rats, the number of UP, LP, MP groups (n = 5) are the same, because the two segments were left in the same rat and the MP was removed for control. Fisher’s exact test. p≤0.05 - significant.

The red and white pulp of the spleen fragments examined were generally well preserved. The lymphatic follicles, number of lymphocytes and sinusoids, cell proliferation, number of vessels were within normal parameters. Minimal (mild) necrosis (which affects less than 33% of the area observed under the microscope) occurred in five cases in the upper pole group and five cases in the lower pole (38.4%), a difference without statistical significance. The other cases of the upper and lower poles were unchanged. These changes were not found in the MP and SG groups whose differences in relation to UP and UP were p = 0.058 (Table 5).

**Table 5:** Light microscopic necrosis of the upper pole (UP), lower pole (LW) and middle portion (MP) of the spleen and simulation group (SG) after subtotal splenectomy with removal of the middle portion of the spleen in 15 rats, with preservation of the entire spleen in 5 rats (simulation group).

Group	N	Mild Necrosis Present	Normal	p^1^	p^2^	p^3^	p^4^	p ^5^
UP	13	5 (38.4%)	8 (61.6%)					
LP	13	5 (38.4%)	8 (61.6%)	1.00				
MP	13	0 (0.0%)	13 (100%)		0.058		0.058	
SG	5	0 (0.0%)	5 (100%)			0.058		0.058

UP - Upper Pole, LP - Lower Pole, MP - Middle Portion, SG - Simulation Group.

p1 - UPxUP Group; p2 -UPxMP Group; p3 – Group UPxSG; p4 - Group LPxMP; p5 - LPxSG.

Moderate microscopic necrosis (affecting between 30 and 50% of the splenic area examined under the microscope) occurred in 15.3% of the upper and 30.7% of the lower poles, a difference that is not significant (Table 6). In the MP and SG groups there was no necrosis, so there was no difference in the frequency of necrosis between these two groups, and between these two groups and the UP and LP groups.

**Table 6:** - Moderate microscopic necrosis of the upper and lower poles of the spleen after subtotal splenectomy with removal of the middle portion of the spleen in 13 rats and with preservation of the entire spleen in 5 rats.

Group	n	Moderate Necrosis Present	Normal	p^1^	p^2^	p^3^	p^4^	p ^5^
UP	13	2 (15.3%)	11 (84.6%)	0.658				
LP	13	4 (30.7%)	9 (69.2%)		0.480			
MP	13	0 (0.0%)	13 (100%)			0.112		
SG	5	0 (0.0%)	5 (100%)				1.00	0.53

UP - Upper Pole, LP - Lower Pole, MP - Middle Portion, SG - Simulation Group. p1 - UPxUP Group; p2 - UPxMP Group; p3 - Group UPxSG; p4 - Group LPxMP; p5 -LPxSG.

**Figure 4:**
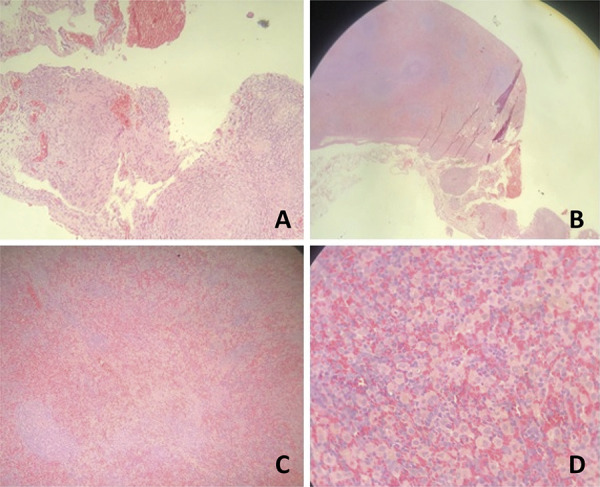
Photomicrograph of the upper pole and the lower pole showing the following aspects: A. HE x10: fibrosis area; B. HE x20: area of fibrosis with foreign body reaction; C. HE x20: necrosis area; D. HE x40: necrosis area with hemosiderophagus. Note that the white pulp and red pulp are preserved.

Minimal (mild) microscopic fibrosis (which affects less than 33% of the area observed under the microscope) occurred in 12 cases in the UP group (92.3%) and 12 cases in the lower pole (92.3%), a difference without statistical significance. There was also no significant difference in fibrosis between the UPxMP, UPxSG, LPxMP, LPxSG groups (Table 7) (Fig. 4).

**Table 7:** Light microscopic fibrosis of the upper and lower poles of the spleen after subtotal splenectomy with removal of the middle portion of the spleen in 13 rats and with preservation of the entire spleen and in 5 rats in the simulation group.

Group	N	Light Fibrosis Present	Normal	p^1^	p^2^	p^3^	p^4^	p^5^
UP	13	12 (92.3%)	1 (7.69%)					
LP	13	12 (92.3%)	1 (7.69%)	1.00				
MP	13	13 (100%)	0 (0.00%)		1.00		1.00	
SG	5	4 (80.0%)	1 (20%)			0.49		0.49

UP - Upper Pole, LP - Lower Pole, MP - Middle Portion, SG - Simulation Group. p1 - UPxUP Group; p2 -UPxMP Group; p3 - Group UPxSG; p4 - Group LPxMP; p5 - LPxSG.

## Discussion

Conservative surgery on the spleen has been increasingly improved due to existing technology.

This study compared the viability of the upper pole and the lower pole of the spleen of rats, both preserved in the same animal (first control), in addition to the study presenting a second control (medium portion removed) and a third control (simulation), to evaluate the viability of splenic remnants.

Our results showed that there was no statistically significant difference between the viability of the upper pole and the lower pole of the spleen of rats, when compared to each other and when compared to the middle portion and the simulation group. In one rat only, in which both poles were left, a lower pole was not found, which speaks in favor of autolysis of this segment due to poor blood supply. This may have been due to the lack of initial deficient irrigation to the pole, or it may have been due to the inadvertent section of a thin transparent membrane that joins the stomach to the spleen, which enables the rotation of this segment and consequently the vascular torsion with segmental devascularization. However, in one study, the section of the gastroesplenic peritoneal membrane did not change the viability of the lower pole^15^. However, this result was reported in only one study, requiring posterior confirmation on the influence of the membrane section on the viability of the splenic remnant. Aside from this case, the other poles (upper and lower) were macroscopically viable, that is, they presented color, texture, consistency and size maintained in accordance with the original spleen (medium portion removed) and with the simulation group.

When comparing the initial length of the lower pole with the final length, it is observed that there was no significant change. The same occurred with the width and thickness. These results go against those of a study that shows that the lower pole grows over time^16^.

It is not known, however, whether the maintenance of the upper pole together with the lower pole may have inhibited the growth of the lower pole. To better clarify this issue, another study could be carried out, separating a group with only the lower pole and another group with only the upper pole. In addition, in this study, one could test the functions of both poles with the control of a group with an intact spleen and a group without a spleen (total splenectomy). Anyway, even though the pole did not increase in size, it was macroscopically viable. On the other hand, the upper pole increased in length, but not in width and thickness. This shows feasibility characteristic. However, the lower pole grew in width more than the upper pole. This also shows the feasibility characteristic.

The microscopic findings confirmed the viability of both poles, with the presence of white pulp (lymphoid follicles and their components), red pulp, vascularity and cellularity specific to the organ, in addition to possible inflammatory processes. Occasionally, there was a sparse area of fibrosis and sparse necrosis that was not representative. No abscesses or areas of important fibrosis and necrosis were found. Finally, from a macro and microscopic point of view, both splenic poles remained viable.

This study has the following limitations:

1. The number of rats was small. Larger samples could show other results.
2. It is not absolutely certain whether or not there was a section of the gastroesplenic membrane that seems to fix the spleen to the stomach and, thus, prevent the rotation of the splenic pedicle, since it does not use a surgical microscope or magnifying glass during the surgical procedure. Another study, contemplating the use of this equipment, with section and non-section of this membrane, could better clarify this issue and verify the effect of membrane preservation on splenic viability.
3. This is an experimental study and its results should be viewed with caution when transposing to human beings.

## Conclusion

The upper and lower poles of the spleen after subtotal splenectomy showed no statistically significant difference in terms of viability, with both poles remaining viable, the lower in 90% of cases and the upper in 100% of cases, a difference that is not significant.
